# Brain Removal With an Occipital Hinge Preserving Overlying Anatomy

**DOI:** 10.7759/cureus.72377

**Published:** 2024-10-25

**Authors:** Mario Loomis, Jason Nikirk, Teresa Loomis, Dennis Wooten, George Prada III

**Affiliations:** 1 Department of Clinical Anatomy, Sam Houston State University College of Osteopathic Medicine, Conroe, USA

**Keywords:** brain removal, cranial nerves, gross anatomy, occipital hinge, suboccipital triangle

## Abstract

Brain removal during a gross anatomy course in medical school serves multiple purposes. It allows for the teaching of cranial vault anatomy, as well as the external brain, cranial nerves, and cerebral vasculature. Techniques to remove the brain while preserving these delicate structures generally damage the overlying anatomy of the dura and suboccipital triangle. Even though brain removal usually comes later in most gross anatomy courses, with the suboccipital triangle being taught at the beginning, it is still beneficial to preserve previously dissected structures, not only for use in cumulative practical assessments but also for additional teaching opportunities. Shrinking the availability of time and money has prompted anatomy departments to identify and develop potential areas for shared resources to teach medical students in clerkships, residents, and allied health professionals. The ideal brain removal technique would safely remove the brain, preserving its structure, cranial nerves, and vasculature, while also preserving an intact dura and suboccipital triangle. We present an occipital hinge technique of brain removal that preserves the cranial nerves and vasculature, as well as an intact, in situ dura, without damaging previously dissected suboccipital triangles.

## Introduction

Brains are removed during a gross anatomy course to teach cranial vault anatomy, as well as external brain, cranial nerves, and cerebral vasculature. The classic anterior approach to brain removal during autopsies has been used efficiently and effectively for decades. Following a circular craniotomy and removal of the superior calvarium, the dura is removed in segments, and the cranial nerves are severed sequentially from anterior to posterior, along with the internal carotid arteries, the tentorium cerebelli, the vertebral arteries, and the cervical spinal cord, allowing the brain to roll out of the cranial fossae atraumatically [1]. Difficulties arise when this technique is utilized with embalmed cadavers; however, the brains are already fixed and are not as readily retracted from the posterior fossa as fresh brains are. This can lead to injury to the cranial nerves and cerebral vasculature. A study investigating the preservation of these structures, along with the ease and speed of various brain removal techniques, all of which took about thirty minutes to perform, found that only one out of 18 brains had all 12 cranial nerves bilaterally, and only one had all of the brain's vasculature intact [2]. These deficiencies can be problematic for the teaching of neuroanatomy, which has led others to investigate methods to better preserve brain structures by adding an occipital wedge and a cervical spine opening. These additions led to the preservation of all cranial nerves and vasculature with about a 10-minute increase in dissection time [3,4]. The ultimate in the preservation of neural structures is the en bloc removal of the central nervous system, a very time-consuming and laborious process, which has been found to be helpful by a large majority of medical students who recommended that it be available to all students prior to clinical rotations [5]. However, all these techniques designed to optimize the preservation of neural structures involve the destruction of overlying soft tissue in the posterior neck, and they do not maintain intact dural relationships between the brain and skull. The entire dura is either removed from the skull or the dura is partially removed in segments. A brain removal technique that not only optimizes the preservation of neural structures but also preserves intact dural anatomy and suboccipital triangle anatomy has not been previously described. Our hypothesis was that the performance of an occipital hinge rather than resection, with no disruption of normal cervical anatomy, would lead to complete preservation of cranial nerves and vasculature, along with an intact dura, without sacrificing the delicate structures of the suboccipital triangle, an area of clinical importance regarding surgery in the region.

The cadaver utilized in this study was obtained from the McGovern Medical School Willed Body Program in Houston, Texas. The Sam Houston State University IRB ruled this research as exempt, IRB# 2023-118.

## Technical report

**Figure 1:**
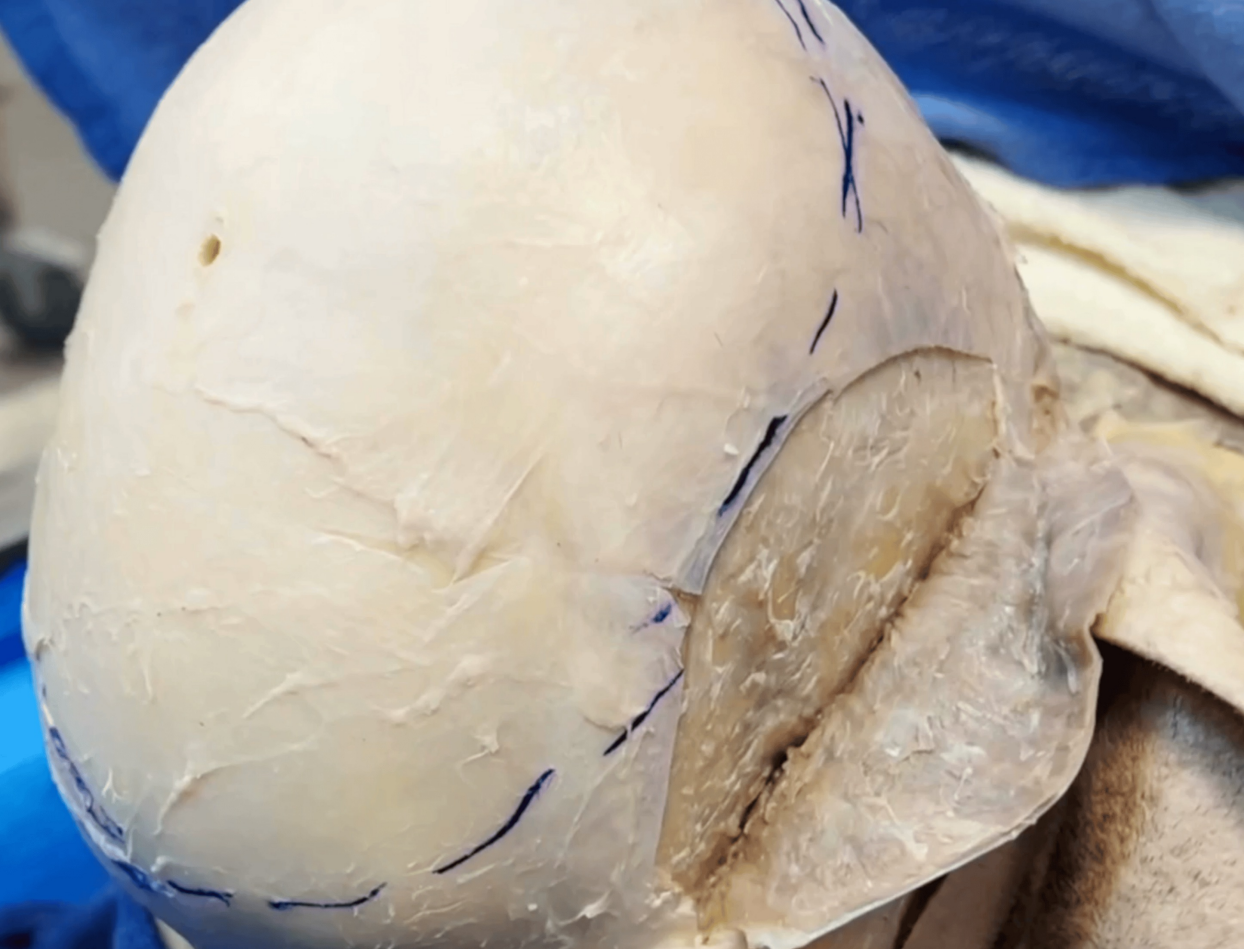
Initial shallow calvarial cut An initial shallow cut is designed to allow for more reliable preservation of dural integrity.

The entire brain removal is performed with the cadaver in the prone position. The scalp and temporalis layers are reflected as with any brain removal technique, and the craniotomy begins. A shallower craniotomy is designed to ensure separation of the periosteum from the calvarium without tearing the dura (Figure 1).

**Figure 2:**
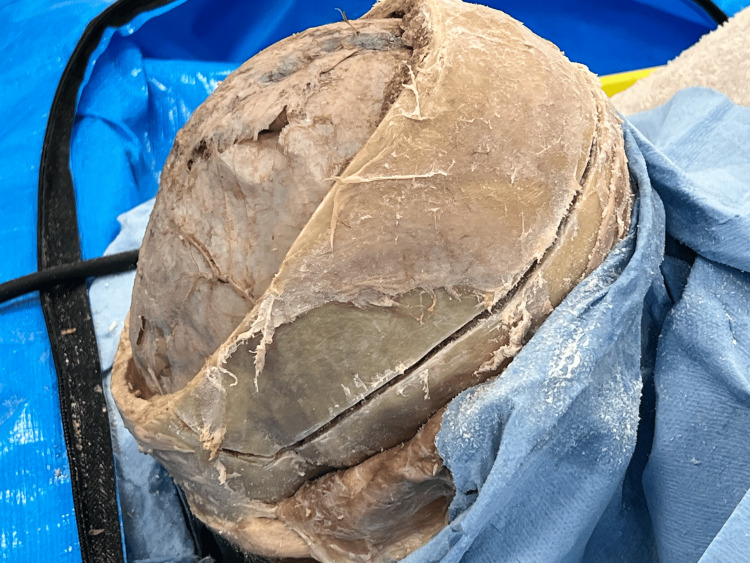
Removal of additional strip Under direct vision, the periosteal dura is elevated off additional calvarium to be removed. The target is to remove calvarium down to the level of the pterion in the anterolateral region and the transverse dural sinus posteriorly. When using the saw, a metal retractor is placed between the dura and the skull to prevent incidental injury to the brain.

Once this cap of calvarium is removed, the dura is separated from the skull under direct vision from the temporal regions to the occipital region. An additional strip of calvarium is then cut for removal, incorporating the pterion anteriorly and extending the occipital cut to just below the transverse dural sinus (Figure 2).

**Figure 3:**
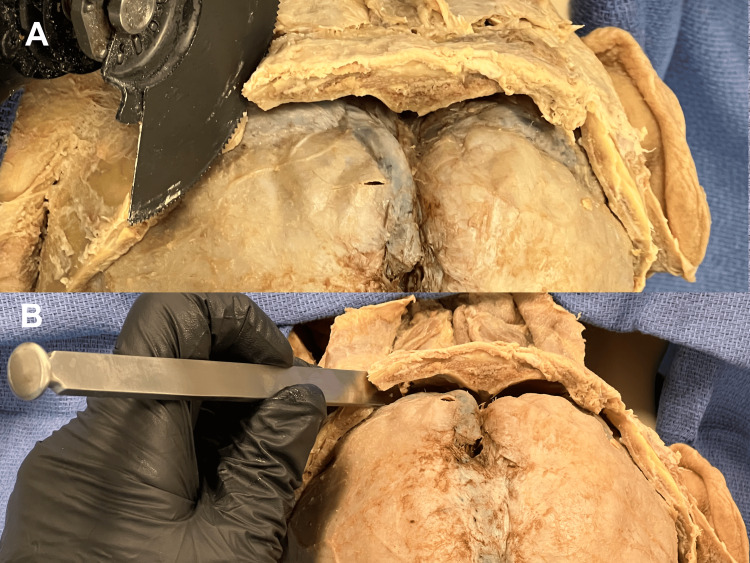
Cutting the occipital hinge (A) A reciprocating saw is used to begin the osteotomies, extending 1-2 cm so as not to disrupt the cervical musculature. (B) An osteotome is then used to continue the fracture line towards the foramen magnum, keeping its edge within the bone so as not to damage the overlying soft tissue.

The periosteum is again elevated off the skull under direct vision, this time down to and into the foramen magnum. Imaginary lines are drawn from the 9:00 and 3:00 positions of the foramen magnum at 45° angles up to the cut edge of the calvarium for the planned occipital hinge. This osteotomy begins with the saw but is completed with an osteotome whose edge is kept within the diploic space of the occipital bone, extending the fracture down to the foramen magnum (Figure 3).

**Figure 4:**
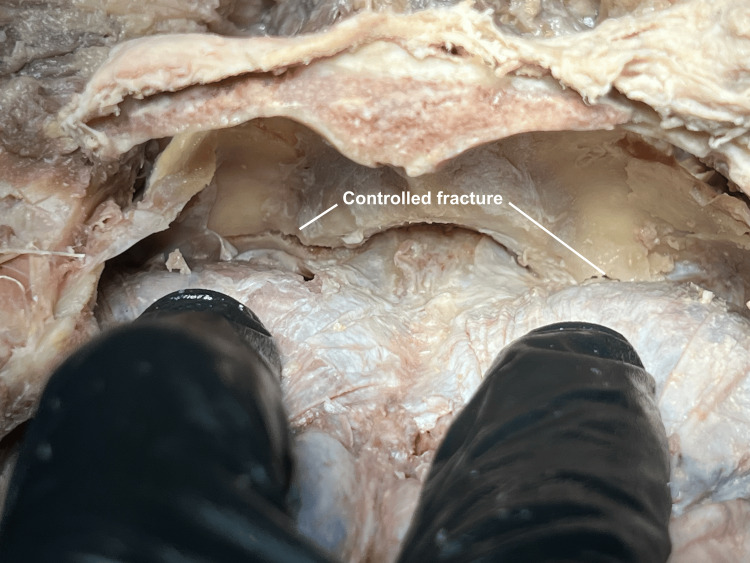
Controlled fracture As the fracture is extended with the osteotome, caudal hinge motion of the occipital wedge is attempted until the fracture is completed into the foramen magnum.

A controlled fracture is then carried out by applying caudal pressure on the occipital wedge segment (Figure 4).

**Figure 5:**
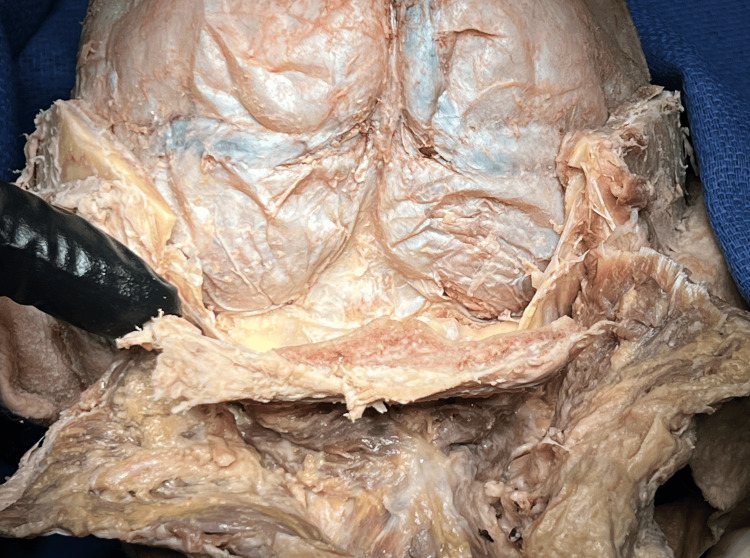
Hinge exposure The hinge allows for excellent visualization of and access to the caudal brain and rostral cervical cord.

The motion of the hinge is optimized by gently rocking the segment back and forth, providing excellent visualization of and access to the caudal brain and cervical spinal cord (Figure 5).

**Figure 6:**
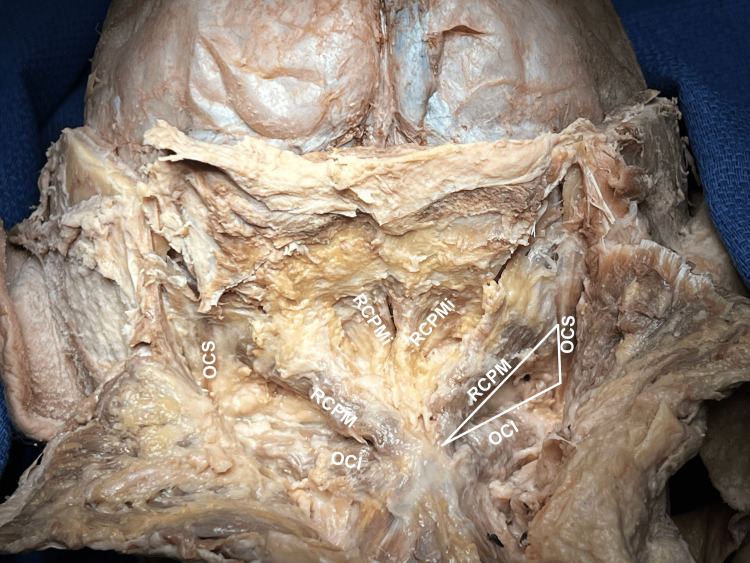
Suboccipital triangle With the hinge returned to its anatomical position, the previously dissected suboccipital triangle is seen to be undamaged. RCPM: rectus capitis posterior major; RCPMi: rectus capitis posterior minor; OCI: obliquus capitis inferior; OCS: obliquus capitis superior.

When the hinge is returned to its anatomical position, the suboccipital triangle is noted to be intact and undamaged (Figure 6).

**Figure 7:**
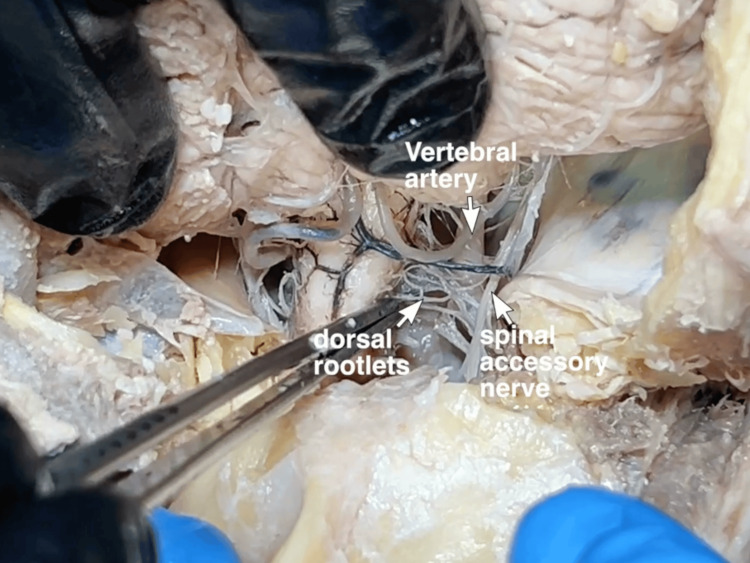
Vertebral artery With the hinge opening the foramen magnum, there is the ease of access to the cervical cord for the division at the level of C2. The vertebral artery can be visualized anterior to the atlantooccipital membrane, the floor of the suboccipital triangle.

**Figure 8:**
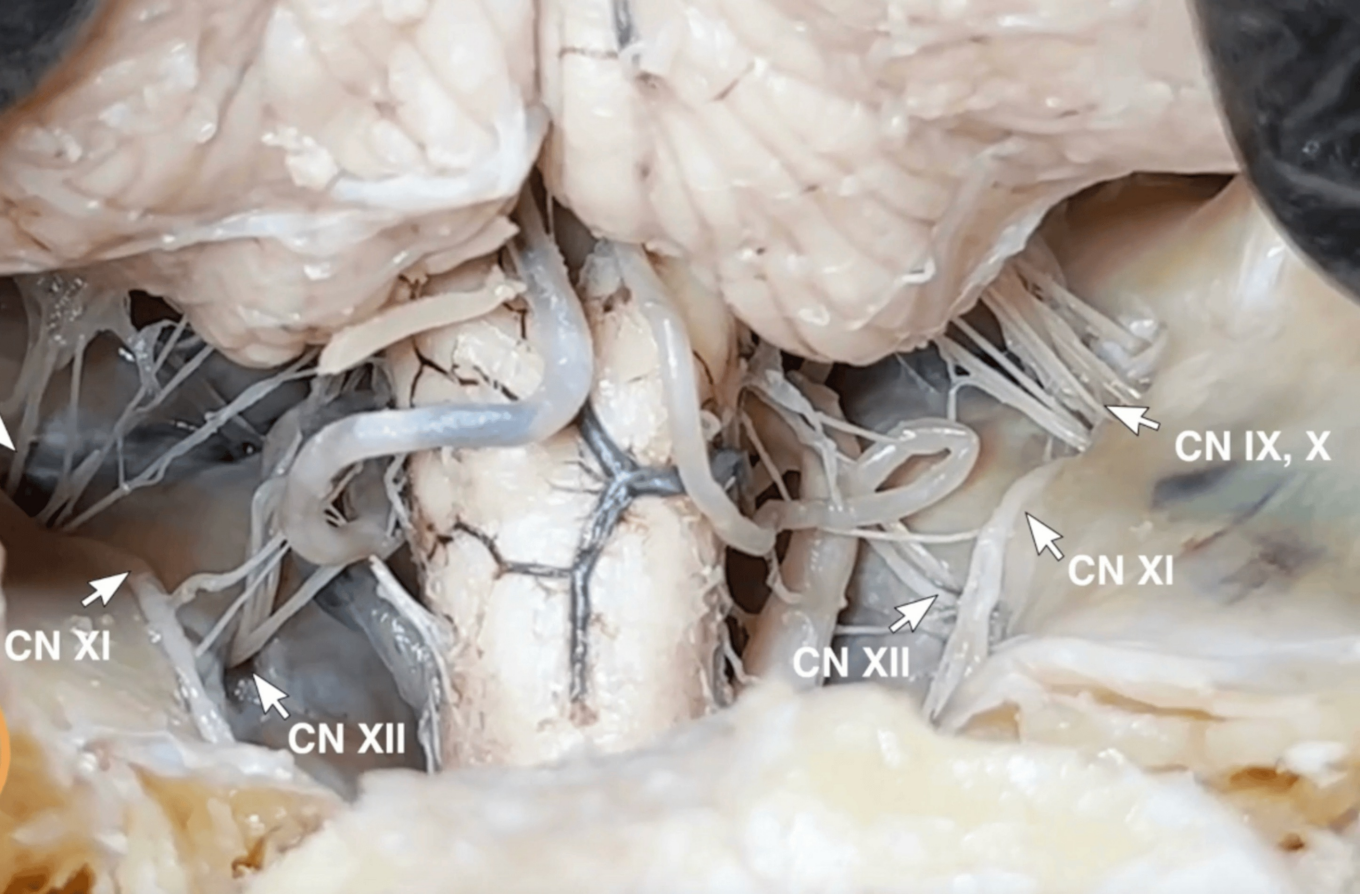
Lateral pontine cistern Approaching from the occipital hinge, the subarachnoid space making up the lateral pontine cistern can be appreciated superior to the jugular foramen and anterior to the cerebellum. Cranial nerves IX, X, and XII are divided under direct vision.

**Figure 9:**
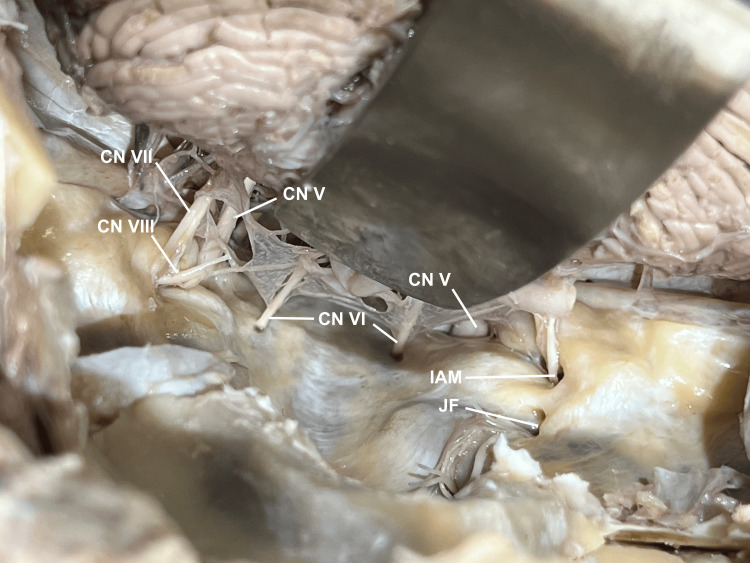
Cerebellopontine angle With the division of cranial nerves IX, X, and XII, the brain is retracted further, and cerebellopontine angle anatomy can be appreciated. The Abducens nerves are seen on the clivus and can be transected leaving an adequate length on the brainstem. CN VII and VIII are visualized entering the internal auditory meatus, and the proximity of CN V can also be appreciated. IAM: internal auditory meatus; JF: jugular foramen.

Through the occipital hinge access, the dura is incised circumferentially, leaving one temporal side attached. The tentorium cerebelli is incised under direct vision, as is the spinal cord at the level of C2. As the brain is gently retracted rostrally, the vertebral arteries are divided along with the cranial nerves, beginning caudally and moving rostrally (Figures 7-9).

**Figure 10:**
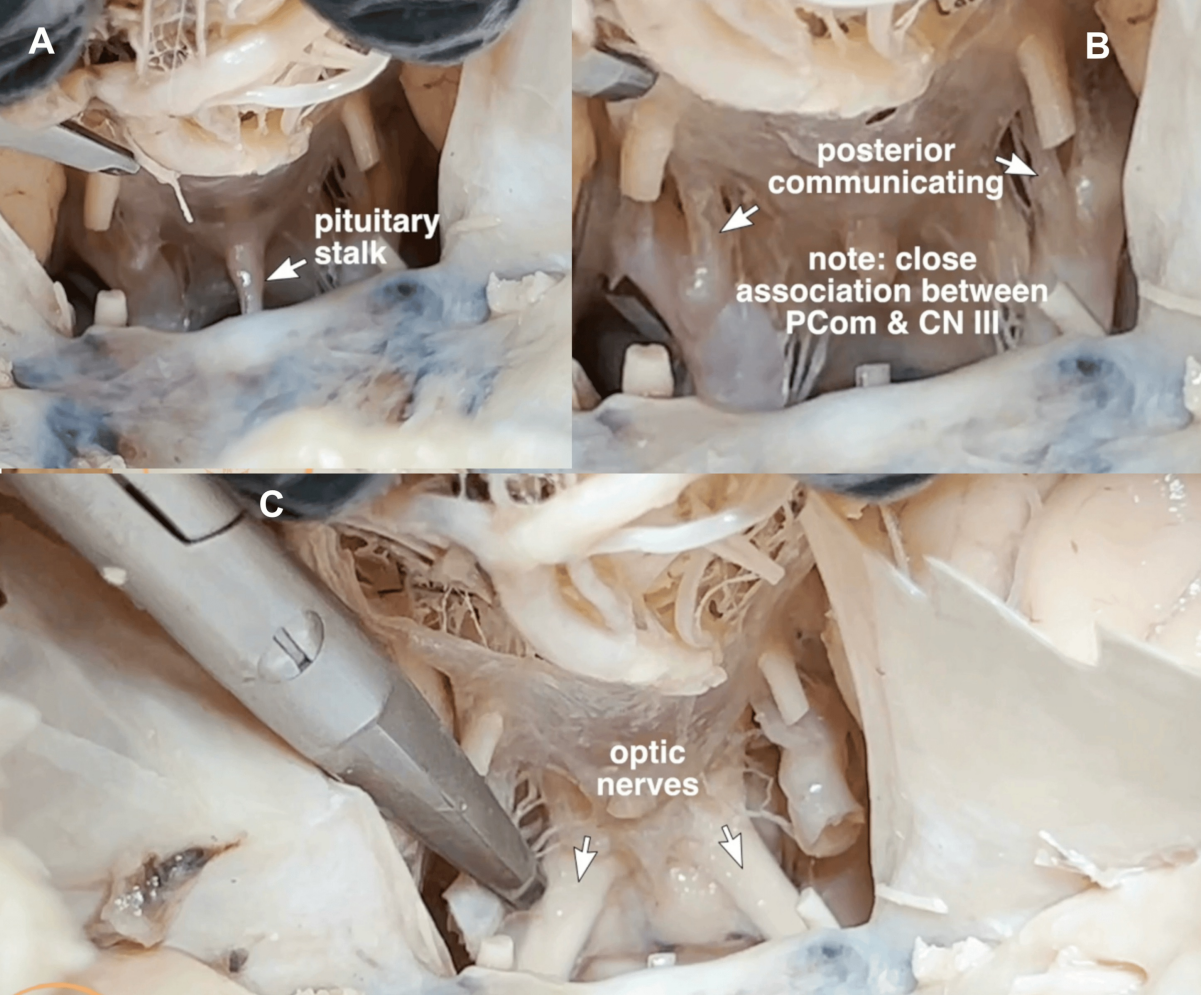
Middle cranial fossa The middle cranial fossa structures are visualized following transection of CN III. (A) The pituitary stalk is clearly seen prior to its division. (B) With additional retraction, the close association between the posterior communicating artery and CN III is appreciated, allowing for an impactful clinical correlation of how ipsilateral mydriasis (pupillary dilation) can signal a life-threatening aneurysm in that artery. (C) With division of the carotid arteries, the relationship between the pituitary gland and the optic chiasm is appreciated, with a visually impactful correlation of how a pituitary tumour can compress crossing nasal retinal fibers in the chiasm leading to bitemporal hemianopia, or loss of peripheral vision.

In the middle cranial fossa, with the division of the oculomotor nerves, the pituitary stalk is clearly visualized, and the close relationship between the oculomotor nerves and the posterior communicating artery can be appreciated. With the transaction of the carotid arteries, the relationship between the pituitary and optic chiasm is likewise appreciated (Figure 10).

## Discussion

The ideal goal for brain removal in an anatomy curriculum is the preservation of both neural and overlying structures in a reasonable amount of time. The occipital wedge technique we describe can be carried out in 45 minutes by an experienced anatomy professor. All cranial nerves and vasculature are preserved, as well as in-situ dura and suboccipital triangle anatomy. The occipital segment hinges upon the posterior atlantooccipital membrane, which is kept intact. Anterior to this membrane, which makes up the floor of the suboccipital triangle, is the suboccipital nerve innervating the muscles of the triangle and the horizontal segment of the third part of the vertebral artery. These anatomical relationships are clinically relevant to surgery in the region during which the vertebral artery is located and mobilized [6].

In addition to the preservation of the suboccipital triangle, the posterior approach through the hinge provides sufficient access to the cervical cord to allow for an effortless division at the level of C2. As the dissection continues rostrally, there is an improved appreciation of posterior cranial fossa anatomy, such as the lateral pontine cistern, a subarachnoid space anterior to the flocculus and between the jugular foramen and internal auditory meatus. Directly visualizing this space can facilitate students' understanding of cerebrospinal fluid flow from the lateral apertures by the flocculus to the pons and ventral brain, continuing over the cerebral hemispheres to the arachnoid granulations. After dividing the glossopharyngeal and vagus nerves and retracting the brain further, there is a clear view of the cerebellopontine angle. The interrelationships within this space can be challenging to appreciate once the brain is removed. Still, during removal (or re-removal after replacing the brain into the dura), they can be visualized and correlated with surgical approaches to treat such entities as cerebellopontine angle tumours, compression of the glossopharyngeal nerve, trigeminal neuralgia, and cerebrovascular aneurysms [7].

**Figure 11:**
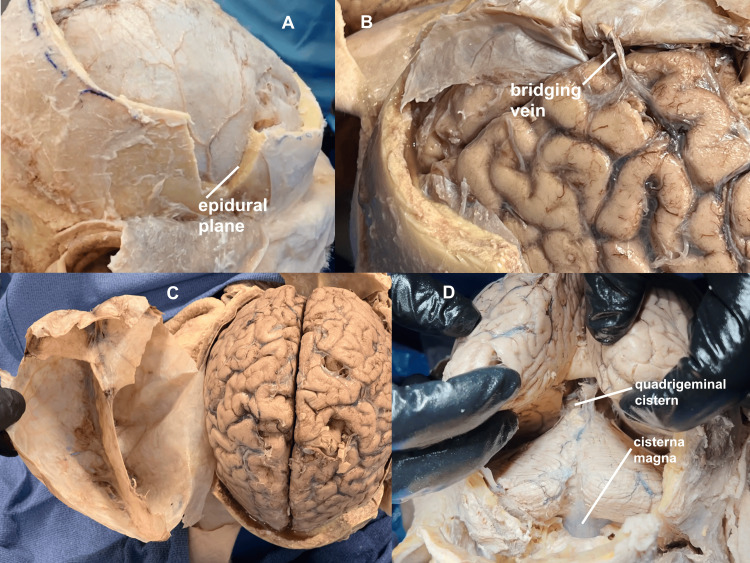
Optimized learning opportunities The technique allows for a number of learning opportunities during the craniotomies. (A) The pterion can be removed individually or with a wide strip of the additional calvarium, demonstrating the middle meningeal artery and the tight epidural plane where the bleeding would accumulate, explaining the time such periosteal elevation would take, hence the lucid interval, as well as the lens or biconvex shape of the epidural haemorrhage on a CT scan. (B) The bridging veins can be appreciated as the source of a subdural bleed, with the broad subdural space explaining the crescent nature of a subdural haematoma on a CT scan. (C) With the dura's integrity maintained, the various dural sinuses can be clearly visualized in three dimensions, associating them with the brain in situ. (D) Prior to breaking through the arachnoid layer, two major CSF cisterns can be appreciated: the Quadrigeminal cistern and the Cisterna magna.

The preservation of an intact and in-situ dura provides a three-dimensional structure to appreciate the pathways of the dural sinuses and other learning opportunities (Figure 11).

The brain can be replaced into the dura, and when students unwrap the brain, they can review the relationships between the calvarium and dura, the middle meningeal artery and epidural bleeds, bridging veins and subdural bleeds, and the subarachnoid layer and subarachnoid bleeds.

Nine second-year students training to be teaching assistants in the upcoming year's anatomy course participated in the occipital hinge technique for brain removal and were asked to compare it to the standard anterior approach they had experienced the year before as first-year students. On a Likert scale, all nine students either agreed or strongly agreed that the novel craniotomy approach allowed for full preservation of the suboccipital triangle, better preservation and visualization of the cranial nerves and cerebral vasculature, better understanding of the posterior cranial fossa and cerebellopontine angle, and a better understanding of the causal relationships behind epidural, subdural, and subarachnoid haematomas.

Anatomy programs can benefit from greater precision in brain removal that preserves overlying structures, allowing for expanded utilization. With prosections that maintain the overlying dura and suboccipital triangles, there are additional audiences who could learn from the generous gift the cadaver donors have made. Anticipating such shared teaching opportunities further supports the unique benefits of cadaver dissection. It can help offset the tendency, which has grown over the past decades, to reduce this precious resource [8].

## Conclusions

The occipital hinge approach provides greater posterior exposure for removal of the brain while preserving cranial nerves and vasculature, an intact in-situ dura, and an undamaged suboccipital triangle. These structures provide additional teaching opportunities for students in allied health programmes, clerkship rotations, and graduate medical education programs. The ability to replace and subsequently unwrap the brain can help students appreciate spaces like the lateral pontine cistern and the relationship between the brain and skull at the cerebellopontine angle.
